# Supplementary material for: Spatial Analysis of Determinants of COVID-19 Vaccine Hesitancy in Portugal
Source: Vaccines (Basel). 2024 Jan 24;12(2):119. doi: 10.3390/vaccines12020119 (PMC10892156; doi:10.3390/vaccines12020119)
Supplement: Supplementary file 1 [file vaccines-12-00119-s001.zip › vaccines-2801349-supplementary.pdf]

Supplementary material: Spatial analysis of determinants of COVID-19 vaccine hesitancy in Portugal

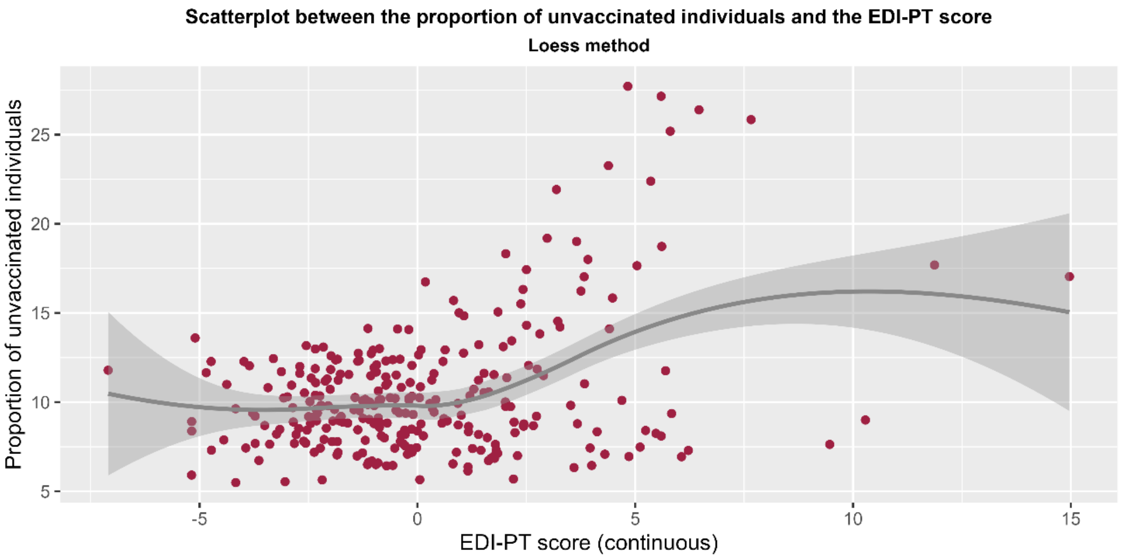

Figure S1. Scatterplot between the proportion of unvaccinated individuals and the EDI-PT score (each dot represents a municipality).

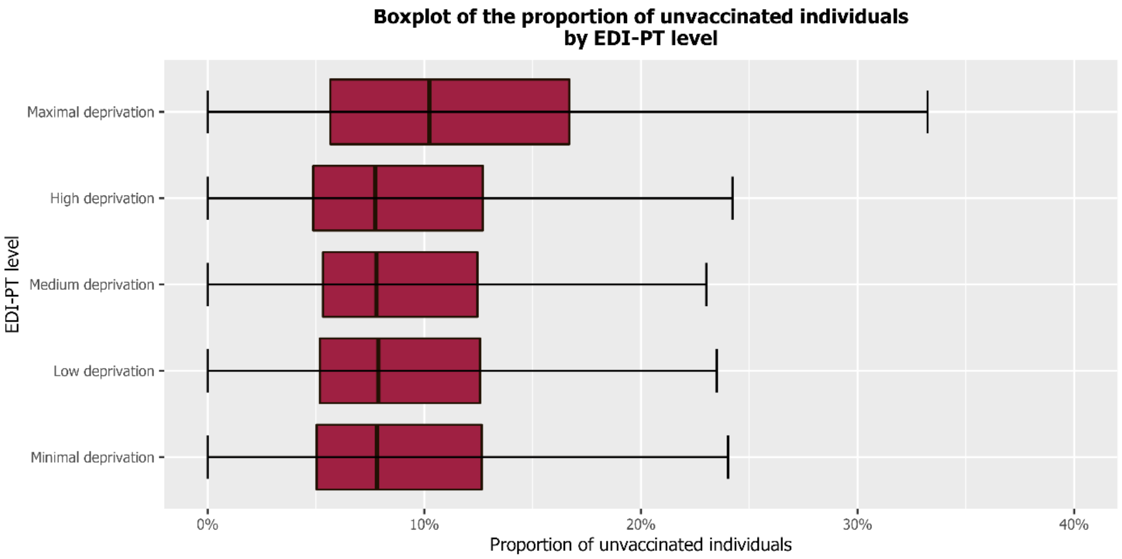

Figure S2. Boxplot of the proportion of unvaccinated individuals by EDI-PT level.

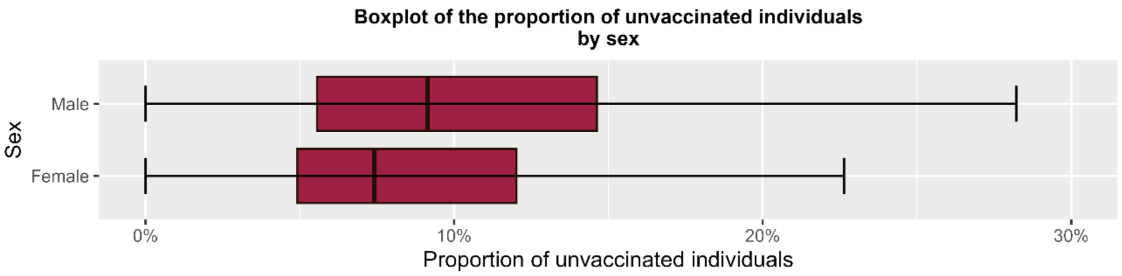

Figure S3. Boxplot of the proportion of unvaccinated individuals by sex.

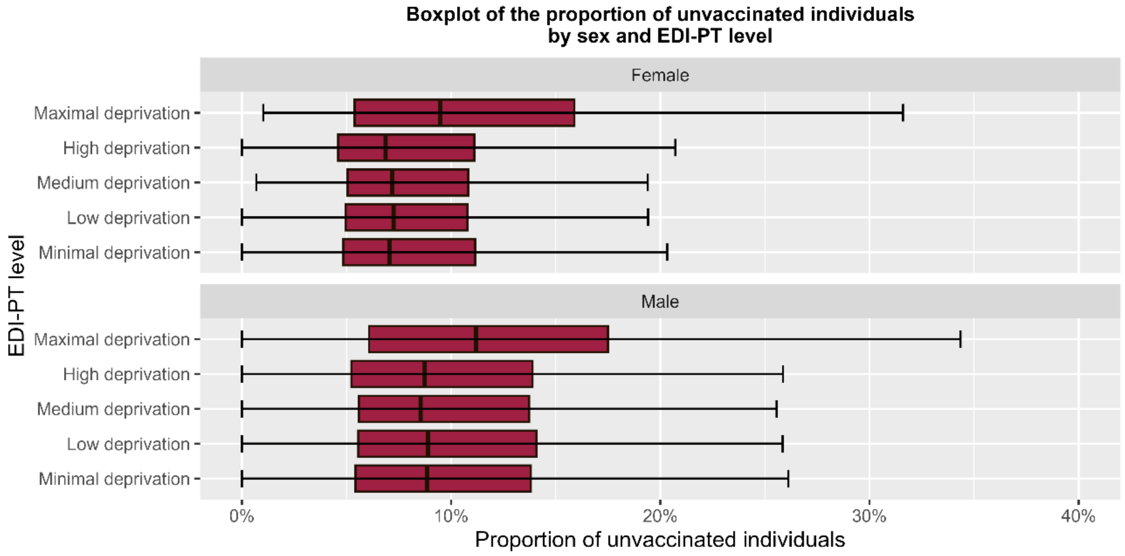

Figure S4. Boxplot of the proportion of unvaccinated individuals by sex and EDI-PT level.

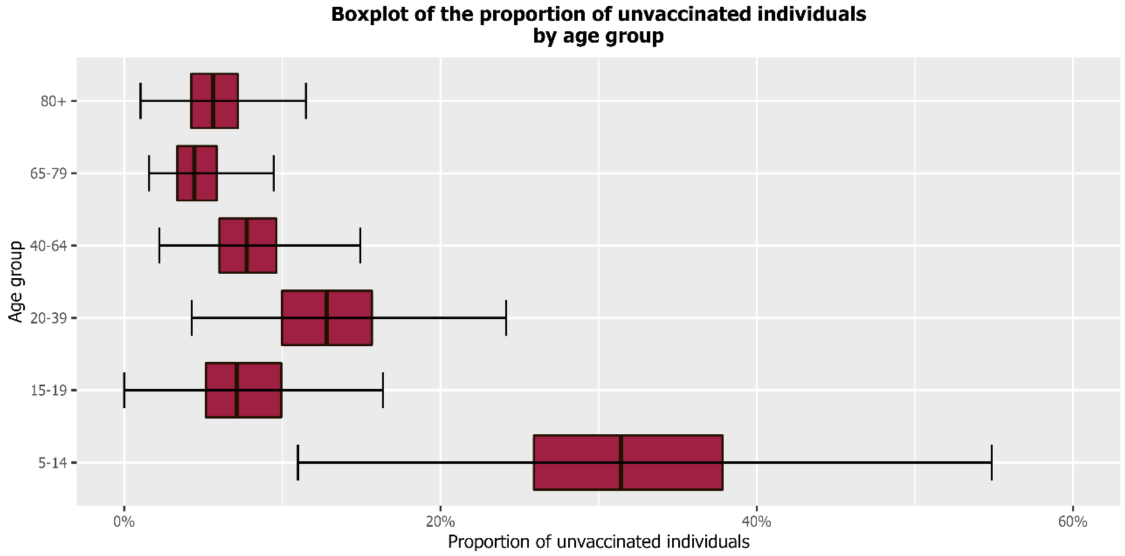

Figure S5. Boxplot of the proportion of unvaccinated individuals by age group.

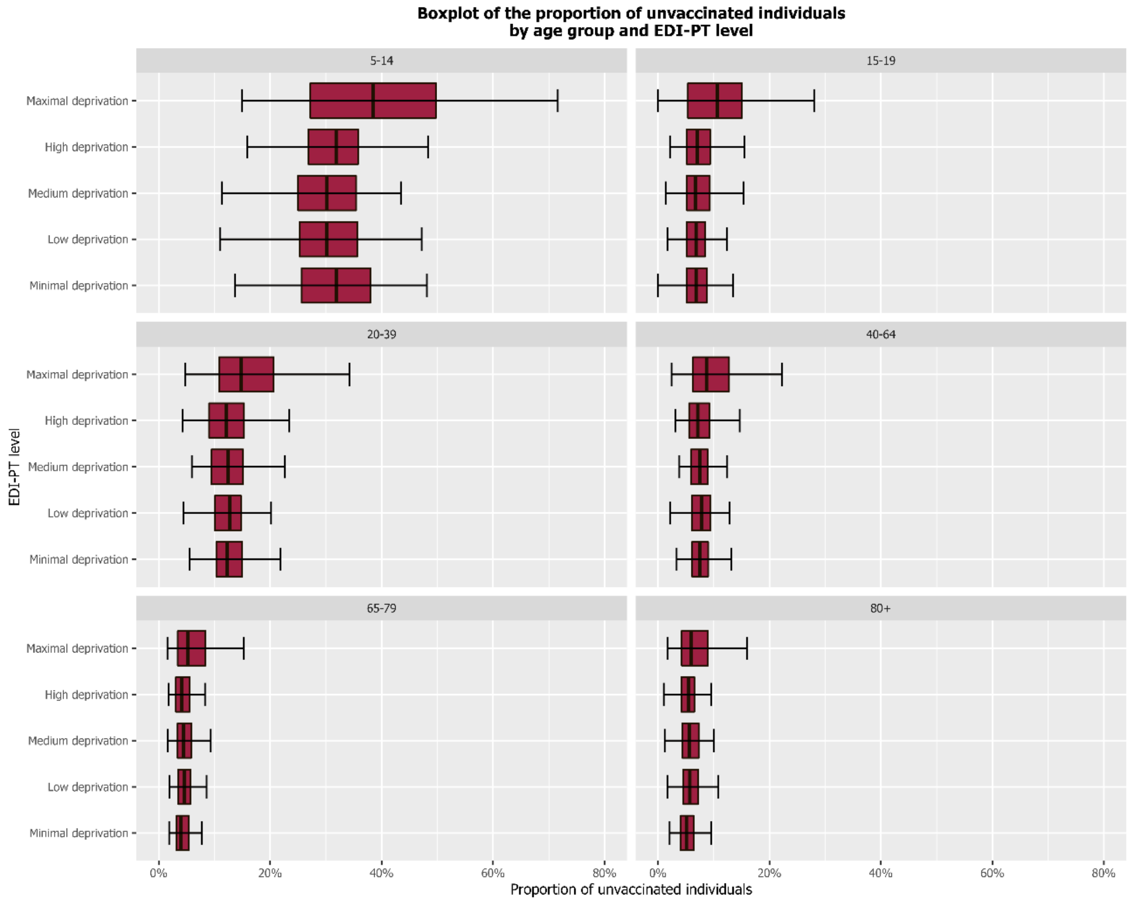

Figure S6. Boxplot of the proportion of unvaccinated individuals by age group and EDI-PT level.

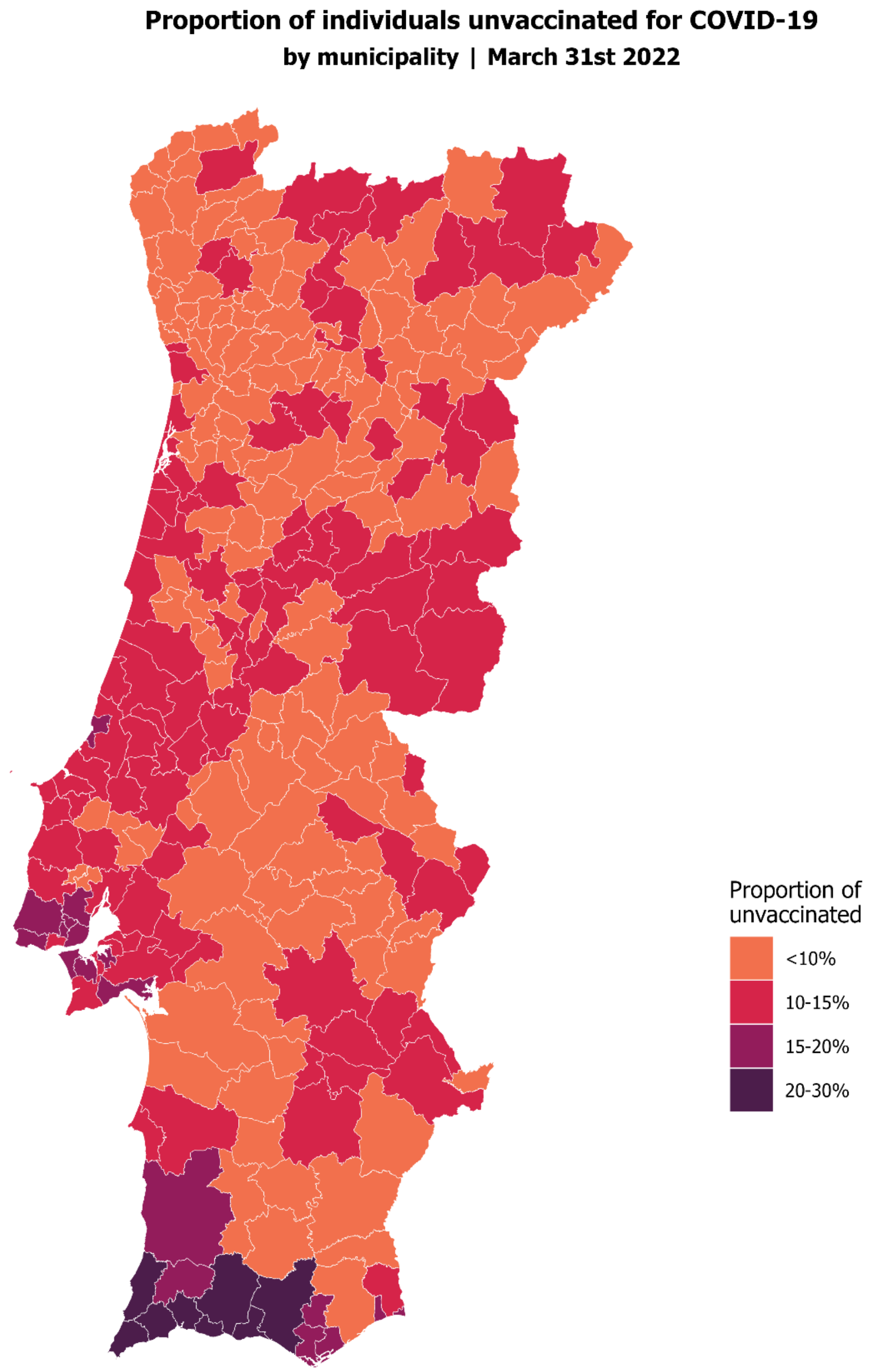

Figure S7. Map of the distribution of the proportion of unvaccinated individuals by municipality.

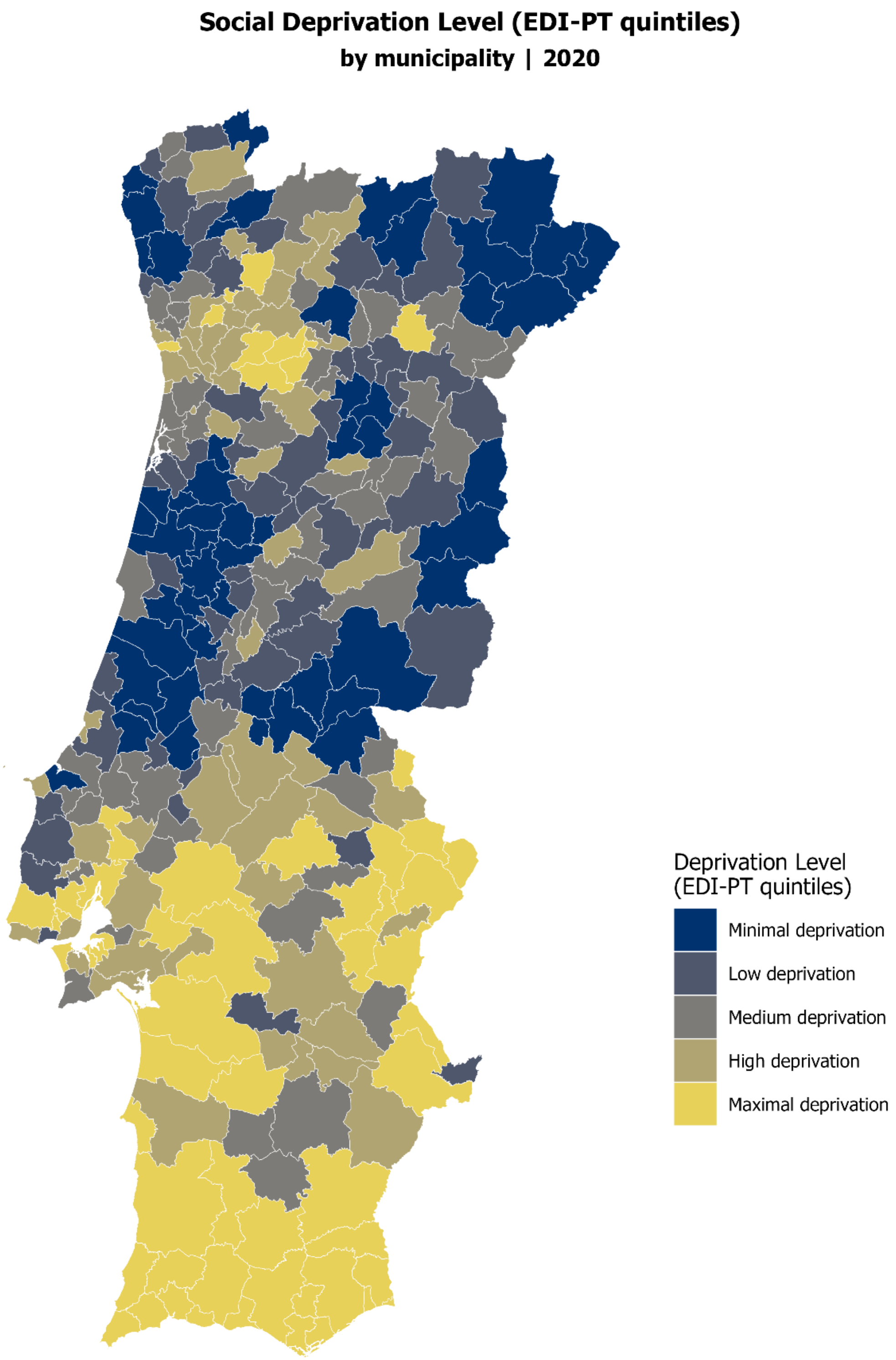

Figure S8. Map of the distribution of the social deprivation levels (EDI-PT quintiles) by municipality.

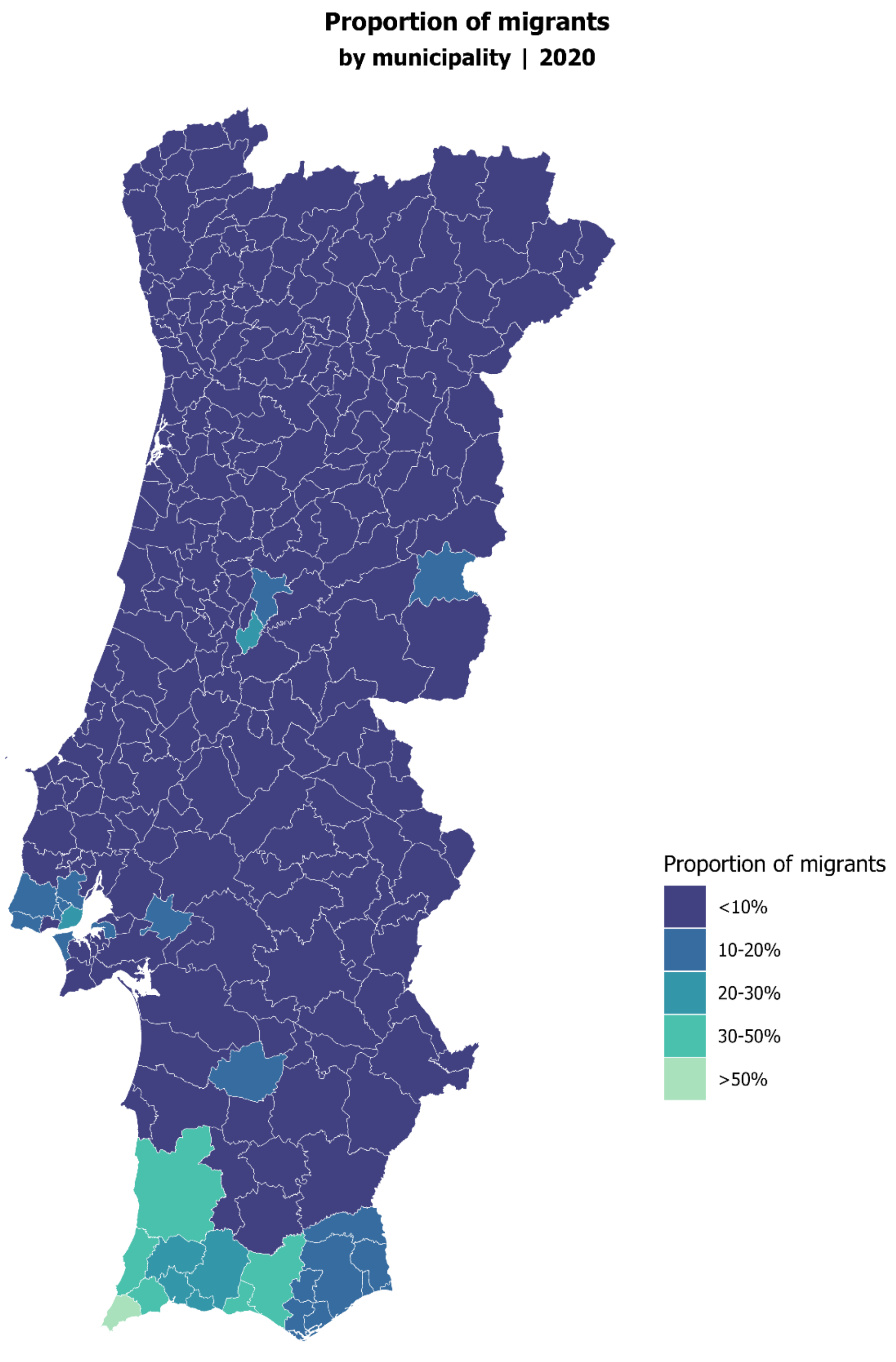

Figure S9. Map of the distribution of the proportion of migrants by municipality.

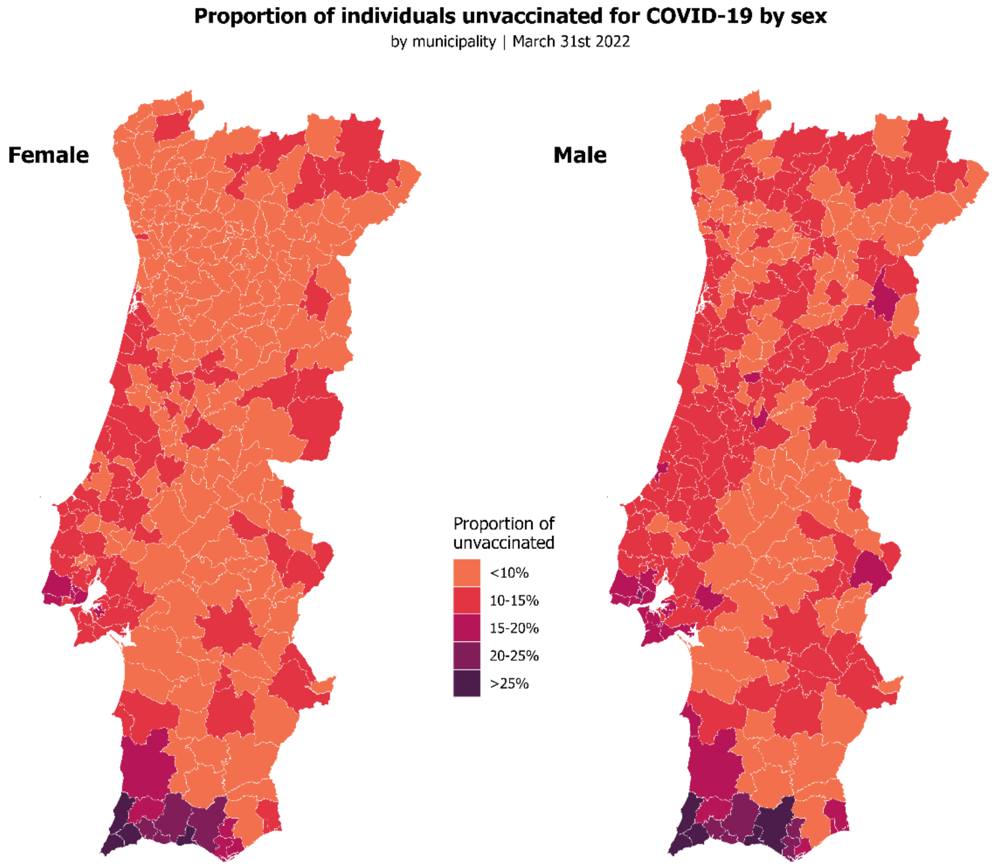

Figure S10. Map of the distribution of the proportion of unvaccinated individuals by sex and municipality.

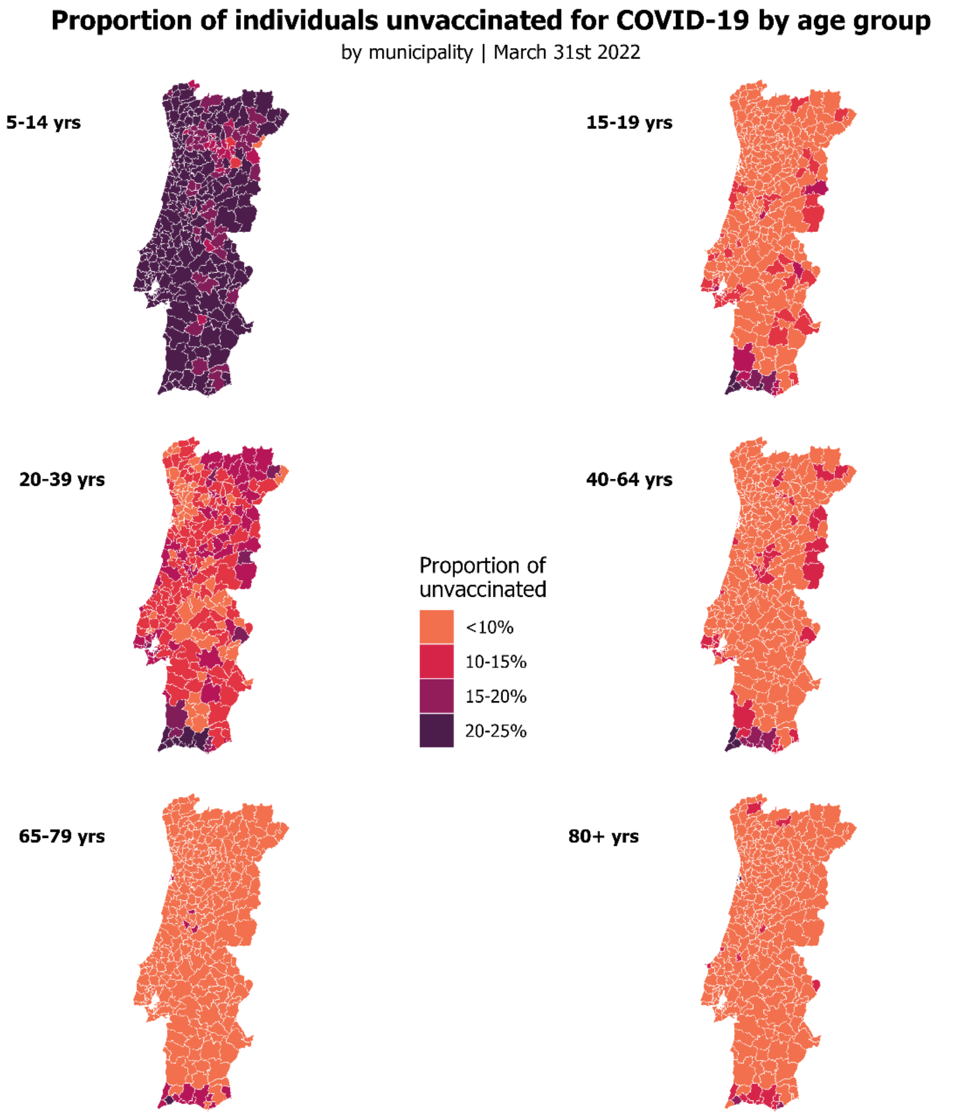

Figure S11. Map of the distribution of the proportion of unvaccinated individuals by age group and municipality.

Table S1. Results from the univariate model with the EDI-PT score.

	Mean	SD	0.025 quantile	0.5 quantile	0.975 quantile
**Intercept**	-0.153	0.046	-0.163	-0.153	-0.144
**EDI-PT score**	0.005	0.006	-0.007	0.005	0.017

Table S2. Results from the univariate model with the EDI-PT quintiles.

	Mean	SD	0.025 quantile	0.5 quantile	0.975 quantile
**Intercept**	-0.205	0.030	-0.263	-0.205	-0.147
**EDI-PT quintile 2**	0.011	0.036	-0.060	0.011	0.081
**EDI-PT quintile 3**	0.067	0.040	-0.011	0.067	0.146
**EDI-PT quintile 4**	0.093	0.044	0.006	0.093	0.180
**EDI-PT quintile 5**	0.085	0.051	-0.016	0.085	0.185

Table S3. Results from the univariate model with the sex (proportion of women).

	Mean	SD	0.025 quantile	0.5 quantile	0.975 quantile
**Intercept**	-1.385	0.734	-2.827	-1.384	0.055
**Prop. women**	0.024	0.014	-0.004	0.024	0.051

Table S4. Results from the univariate model with the 5-14 years age group.

	Mean	SD	0.025 quantile	0.5 quantile	0.975 quantile
**Intercept**	-0.449	0.094	-0.633	-0.449	-0.265
**5-14 yrs**	0.036	0.011	0.014	0.036	0.058

Table S5. Results from the univariate model with the 15-19 years age group.

	Mean	SD	0.025 quantile	0.5 quantile	0.975 quantile
**Intercept**	-0.325	0.010	-0.521	-0.325	-0.130
**15-19 yrs**	0.036	0.021	-0.005	0.036	0.076

Table S6. Results from the univariate model with the 20-39 years age group.

	Mean	SD	0.025 quantile	0.5 quantile	0.975 quantile
**Intercept**	-0.638	0.115	-0.863	-0.638	-0.412
**20-39 yrs**	0.023	0.005	0.012	0.023	0.033

Table S7. Results from the univariate model with the 40-64 years age group.

	Mean	SD	0.025 quantile	0.5 quantile	0.975 quantile
**Intercept**	0.219	0.297	-0.365	0.218	0.800
**40-64 yrs**	-0.010	0.008	-0.026	-0.010	0.006

Table S8. Results from the univariate model with the 65-79 years age group.

	Mean	SD	0.025 quantile	0.5 quantile	0.975 quantile
**Intercept**	0.048	0.099	-0.147	0.048	0.242
**65-79 yrs**	-0.010	0.005	-0.021	-0.010	0.000

Table S9. Results from the univariate model with the 80 years or more age group.

	Mean	SD	0.025 quantile	0.5 quantile	0.975 quantile
**Intercept**	0.007	0.053	-0.097	0.007	0.112
**80+ yrs**	-0.017	0.006	-0.028	-0.017	-0.006

Table S10. Results from the univariate model with the proportion of migrants.

	Mean	SD	0.025 quantile	0.5 quantile	0.975 quantile
**Intercept**	-0.274	0.016	-0.305	-0.274	-0.243
**Prop. migrants**	2.243	0.261	1.729	2.244	2.753

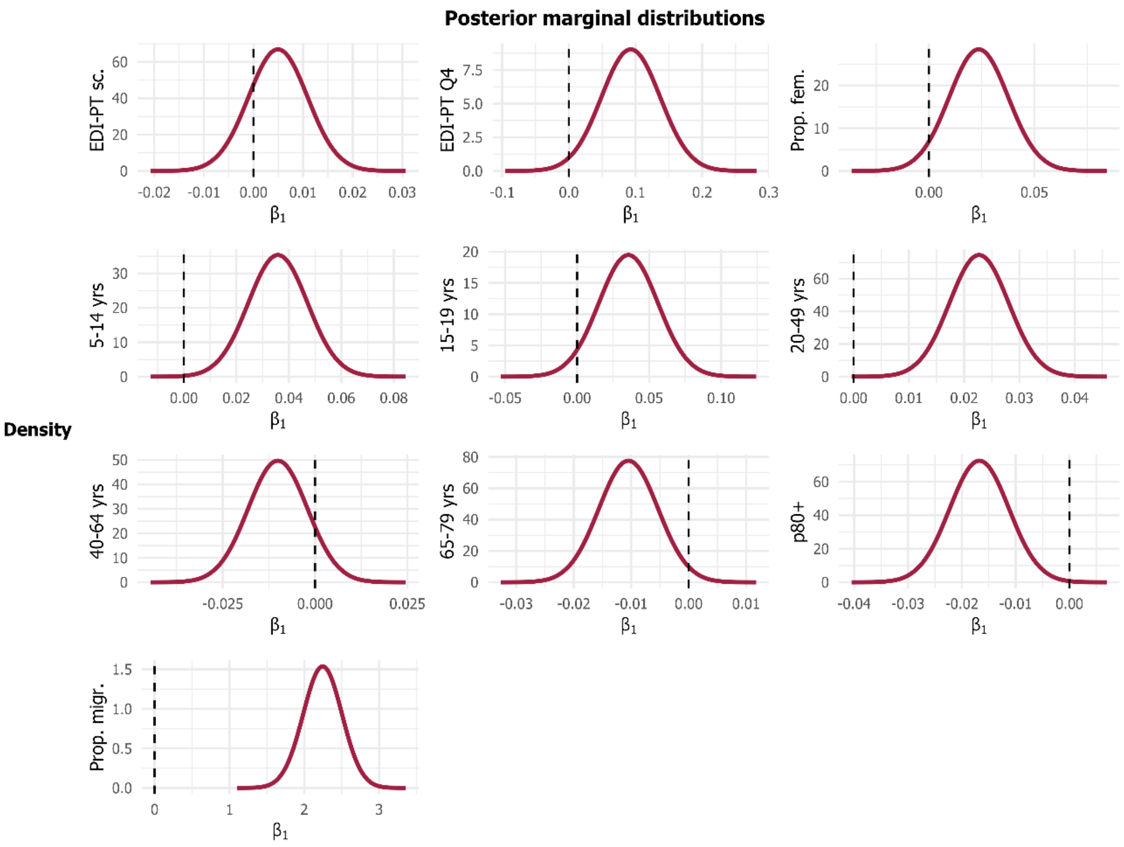

Figure S12. Plots showing the posterior marginal distributions of the univariate models.

Collinearity tests between the EDI-PT and the proportion of migrants

Since the 2018 version of the EDI-PT included an item related to foreign residents, we ran collinearity tests between the EDI-PT variables (score and quintiles) and the proportion of migrants, to exclude the possibility of any interference.

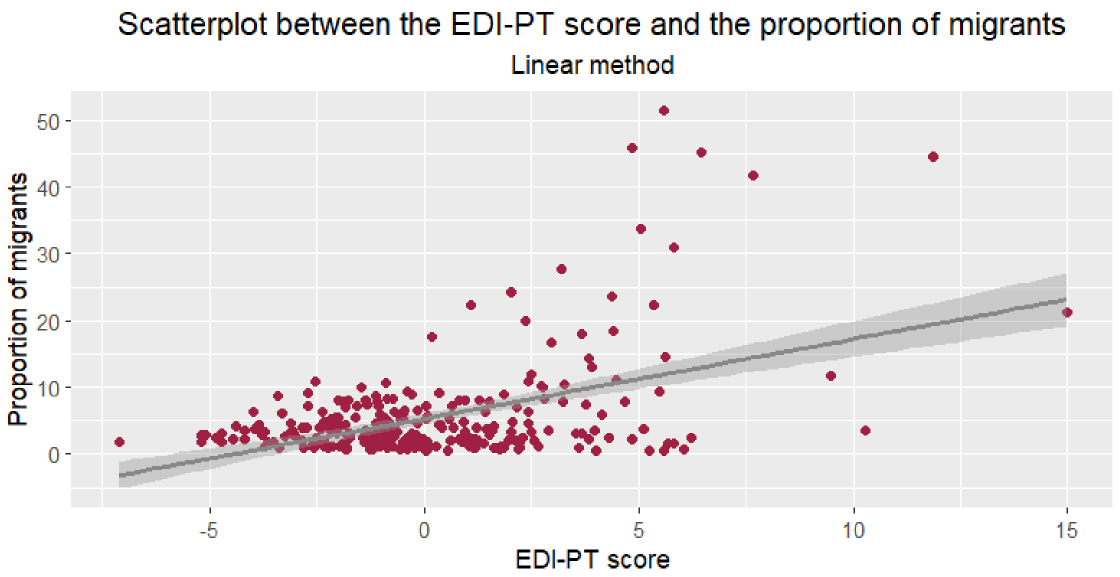

Figure S13. Scatterplot between the EDI-PT score and the proportion of migrants, linear method.

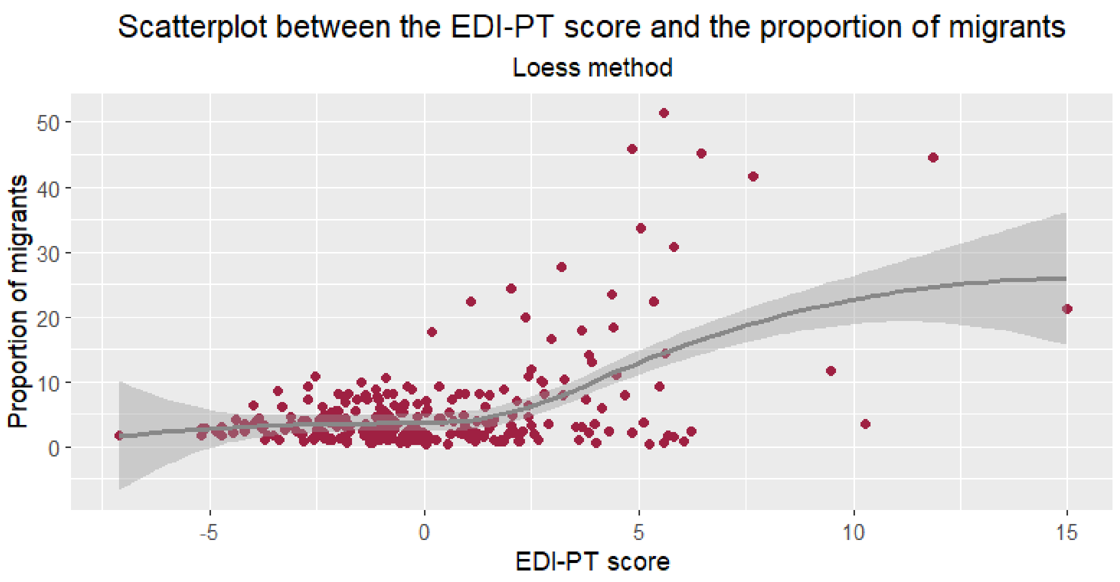

Figure S14. Scatterplot between the EDI-PT score and the proportion of migrants, loess method.

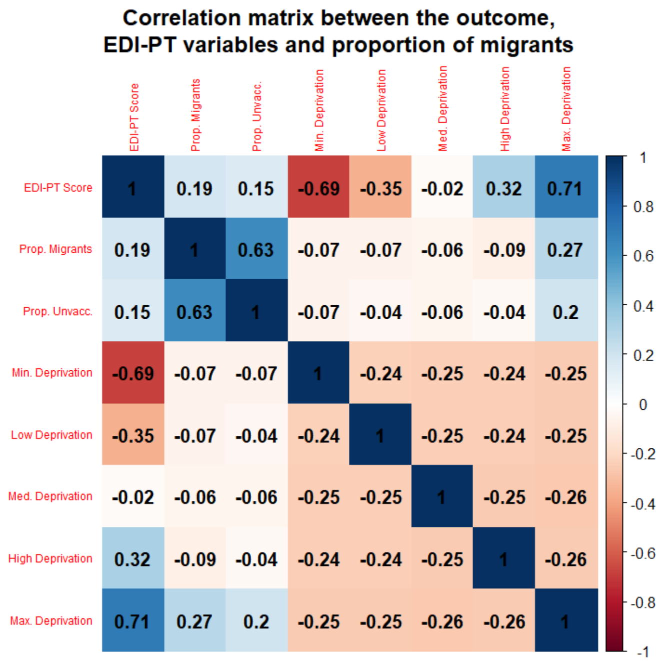

Figure S15. Correlation matrix between the proportion of unvaccinated individuals, EDI-PT variables, and proportion of migrants.

Table S11. Generalized Variance Inflation Factor (GVIF) calculated on a linear model with the proportion of unvaccinated individuals as the dependent variable and the EDI-PT quintiles and the proportion of migrants as the independent variables.

	GVIF	Df	$GVIF^{1/(2*Df)}$
**EDI-PT quintiles**	1.25	4	1.03
**Prop. migrants**	1.25	1	1.12

Notes: GVIF – Generalized Variance Inflation Factor; Df – degrees of freedom

Table S12. Generalized Variance Inflation Factor (GVIF) calculated on a generalized linear model with a *Poisson* distribution with the proportion of unvaccinated individuals as the dependent variable and the EDI-PT quintiles and the proportion of migrants as the independent variables.

	GVIF	Df	$GVIF^{1/(2*Df)}$
**EDI-PT quintiles**	1.44	4	1.05
**Prop. migrants**	1.44	1	1.20

Notes: GVIF – Generalized Variance Inflation Factor; Df – degrees of freedom

Table S13. Generalized Variance Inflation Factor (GVIF) calculated on a generalized linear model with a *Poisson* distribution with the proportion of unvaccinated individuals as the dependent variable and the independent variables used on the multivariate model used in the paper (EDI-PT quintiles, proportion of migrants and 20-39 years age group).

	GVIF	Df	$GVIF^{1/(2*Df)}$
**20-39 yrs**	1.67	1	1.29
**EDI-PT quintiles**	1.57	4	1.06
**Prop. migrants**	1.96	1	1.40

Notes: GVIF – Generalized Variance Inflation Factor; Df – degrees of freedom

Equations from the models included in the paper

(1) Null model without spatial component

$$log(O) = \beta 0 + log(E) + v$$

(2) Null model with spatial component

$$log(O) = \beta 0 + log(E) + u + v$$

(3) Univariate model – EDI-PT score

$$log(O) = \beta 0 + \beta 1 * edis + log(E) + u + v$$

(4) Univariate model – EDI-PT quintiles

$$log(O) = \beta 0 + \beta 1 * ediq + log(E) + u + v$$

(5) Univariate model – Sex

$$log(O) = \beta 0 + \beta 1 * sexo + log(E) + u + v$$

(6) Univariate model – Age groups

5-14 years

$$log(O) = \beta 0 + \beta 1 * ge1 + log(E) + u + v, ge1 = \%\, pop\ 5 - 14$$

15-19 years

$$log(O) = \beta 0 + \beta 1 * ge2 + log(E) + u + v, ge2 = \%\, pop\ 15 - 19$$

20-39 years

$$log(O) = \beta 0 + \beta 1 * ge3 + log(E) + u + v, ge3 = \%\, pop\ 20 - 39$$

40-64 years

$$log(O) = \beta 0 + \beta 1 * ge4 + log(E) + u + v, ge4 = \%\, pop\ 40 - 64$$

65-79 years

$$log(o) = \beta 0 + \beta 1 * ge5 + log(e) + u + v, ge5 = \%\, pop\ 65 - 79$$

80+ years

$$log(o) = \beta 0 + \beta 1 * ge6 + log(e) + u + v, ge6 = \%\, pop\ 80 +$$

(7) Univariate model – Proportion of migrants

$$log(O) = \beta 0 + \beta 1 * pmid + log(E) + u + v$$

(8) Multivariate model

$$log(O) = \beta 0 + \beta 1 * ediq + \beta 2 * ge6 + \beta 3 * pmid + log(E) + u + v$$
